# Different Clinicoradiological Characteristics of Posterior Reversible Encephalopathy Syndrome in Pediatric Oncology and Post-Bone Marrow Transplantation Cases: A Retrospective Study

**DOI:** 10.3389/fneur.2022.836033

**Published:** 2022-05-06

**Authors:** Hwazen Shash, Saad Aldaama, Hala Omer, Sameera Alafghani

**Affiliations:** ^1^College of Medicine, Imam Abdulrahman Bin Faisal University, Dammam, Saudi Arabia; ^2^Department of Pediatrics, King Fahad Hospital of the University, Al-Khobar, Saudi Arabia; ^3^Department of Pediatric Hematology-Oncology and Stem Cell Transplantation, King Fahad Specialist Hospital, Dammam, Saudi Arabia; ^4^Department of Pediatric Hematology-Oncology and Stem Cell Transplantation, King Faisal Specialist Hospital & Research Center, Riyadh, Saudi Arabia

**Keywords:** posterior leukoencephalopathy syndrome, cyclosporine, bone marrow transplantation, hypomagnesemia, systematic hypertension, risk factors, immunosuppression

## Abstract

Posterior reversible encephalopathy syndrome (PRES) is receiving increasing recognition in pediatrics. However, comparisons between PRES in pediatric oncology and post-bone marrow transplantation (BMT) are lacking. Therefore, we aimed to describe the risk factors and clinical and radiological features of PRES and investigate the differences between PRES in pediatric oncology and post-BMT. The PRES data of 13 patients from our center were combined with those of 217 cases from the PubMed, Scopus, and Web of Science databases. The patients were divided into either an oncology or a post-BMT group. We included 230 patients in the analysis, 26.1% of whom belonged to the post-BMT group. Oncology patients developed PRES at a younger age (*p* = 0.010) and were more likely to develop encephalopathy (*p* = 0.004). Systemic hypertension (S-HTN) preceding PRES occurred in 43.5% (66/154) of patients. Post-BMT patients were more likely to have S-HTN (*p* = 0.003). Cyclosporine levels were detected in 37 patients; 40.5% had supra-therapeutic levels. The radiological findings were atypical in 74.3% of patients, and delayed repeated imaging increased the occurrence of resolution (*p* = 0.004). Sixteen (7%) patients developed PRES recurrence after a median of 8 weeks, with the between-group difference being non-significant. Oncology patients were more likely to develop chronic epilepsy, while BMT patients were more likely to develop rare neurologic abnormalities (*p* < 0.001). In conclusion, atypical clinical presentation and imaging findings should not hinder the diagnosis of PRES. S-HTN is a risk factor, particularly in post-BMT patients. Supra-therapeutic levels of cyclosporine and previous exposure to immunosuppression did not increase the risk of recurrence.

## Introduction

Posterior reversible encephalopathy syndrome (PRES) is a distinctive clinicoradiological disorder with acute neurological symptoms (seizures, headache, altered mental status, and visual impairment) and a characteristic parieto-occipital predominant pattern of vasogenic brain edema (1–3). PRES presents with heterogeneous etiologies, clinical presentations, and imaging characteristics across various age groups (4). Although PRES is the established term, its definition remains controversial because of the detection of vasogenic edema outside the parieto-occipital region and the risk of neurologic impairment (5). PRES is a neurological emergency requiring early diagnosis and aggressive management to achieve reversibility and favorable long-term outcomes (6).

The exact pathophysiology of PRES is unclear; however, the two main hypotheses include the vasogenic and cytotoxic theories, along with the more recent immunogenic and neuropeptide theories (2, 3, 7). The vasogenic theory suggests that an increase in systemic blood pressure leads to compromised cerebral autoregulation followed by hyperperfusion, endothelial injury, blood-brain barrier breakdown, and secondary vasogenic edema (7). The reduced sympathetic innervation in the posterior circulation seems to make the posterior areas of the cerebral hemispheres particularly susceptible (2, 3). Children are more vulnerable to cerebrovascular dysfunction as a result of systemic hypertension (S-HTN) due to the narrower range of autoregulation in cerebral blood flow (8). Hypertension (HTN) in patients on chemotherapy may be a side effect of the chemotherapy agent used, such as corticosteroids and cyclosporin A (9, 10). The cytotoxic theory suggests that circulating toxins, such as chemotherapy agents (e.g., vincristine, intrathecal methotrexate) or immunosuppressants (IST) trigger endothelial dysfunction (2). The endothelial dysfunction could initiate vascular leakage, edema formation, and endothelial activation, resulting in the release of immunogenic and vasoactive substances (2). Vasoconstrictive agents are assumed to mediate cerebral vasospasm, subsequent disruption of blood-brain barrier, local hypoperfusion, and vasogenic edema (2). In this theory, blood pressure elevations are a consequence of PRES rather than a causation (2). Given the heterogeneousness of underlying diseases and toxic triggers, PRES in oncology and post-BMT is likely to be multifactorial, with a capillary leak from high blood pressure integrated with direct vascular endothelium damage (7, 11).

Numerous well-defined risk factors of PRES exist in pediatric oncology patients and in those who have undergone bone marrow transplantation (BMT), including chemotherapy, corticosteroids, ISTs, comorbid HTN, and renal dysfunction (2, 3, 12). An increase in the index of suspicion and utilization of advanced neuroimaging have contributed to the rising numbers of PRES diagnoses (2, 3, 13). However, most evidence for the characterization of this syndrome has been obtained from retrospective case reports and case series focusing on particular groups of patients, with no prospective randomized trials accurately determining the risk factors. The available studies mainly examine cohorts of oncology and post-BMT patients, either separately or in combination, but the two groups have rarely been compared. This study describes the clinicoradiological characteristics of PRES in pediatric oncology and post-BMT groups based on a large retrospective sample comprising patients from our hospital and from reports indexed on PubMed, Scopus, and Web of Science. Additionally, we aimed to describe the risk factors contributing to the development of PRES and investigate the differences between patients with oncologic diseases and those who are either undergoing or have completed BMT.

## Materials and Methods

### Data Collection

The study was conducted in accordance with the tenets of the Declaration of Helsinki and was approved by the institutional review board of King Fahad Specialist Hospital in Dammam (IRB-ONC0312). The requirement for informed consent was waived because of the retrospective nature of the study. The PRES diagnostic criteria have been previously described (3) and include the presence of neurological symptoms such as seizures, headache, visual symptoms, abnormal level of consciousness, and hyperintense white matter lesions on T2-weighted imaging. We retrospectively collected patient data as follows: firstly, we searched the medical records of patients with PRES admitted to our hospital between September 1, 2010 and December 31, 2020. All patients aged <18 years who had oncologic diseases or had undergone BMT and met the diagnostic criteria were included. Secondly, we searched the PubMed (RRID: SCR_004166), Scopus, and Web of Science databases for all articles published from January 1, 1996, to December 31, 2020, using the keywords “posterior reversible encephalopathy syndrome” OR “reversible posterior leukoencephalopathy syndrome” AND one of the following: “children,” “pediatric,” “cancer,” and “chemotherapy.” Additionally, we performed a thorough review of the extracted articles. We included patients aged <18 years with hematologic or solid tumors, or post-BMT patients for benign or malignant disorders. Only papers in English that reported the data of individual cases were included, such as details on the treatment administered in the 2 weeks preceding PRES and on the affected brain regions, as evidenced by magnetic resonance imaging (MRI). Papers on oncological and non-oncological diseases were excluded if there was no explicit discussion of individual cases. We also excluded patients with hematologic disorders not treated with BMT, those with central nervous system (CNS) malignancies, and those who had undergone CNS surgery, as well as papers that strictly mentioned suspected offending drugs. Finally, we excluded patients who had undergone BMT with thrombotic microangiopathy.

We collected information on sex, age, underlying diseases, medication administered within 2 weeks prior to PRES occurrence, clinical manifestations, presence of systemic hypertension (S-HTN) in the days preceding PRES, presence of acute blood pressure (BP) elevation during the episode, neuroimaging at presentation and follow-up, PRES recurrence, neurologic status, and outcome. We divided the patients into two groups: oncology patients with newly diagnosed malignancy or on active therapy, and patients who had undergone BMT for benign or malignant disorders. We defined the imaging findings as typical, if there was involvement of the parietal and occipital regions alone, and atypical, if typical T2 changes were observed in other brain areas with or without the involvement of the parieto-occipital region.

### Statistical Analysis

Data from clinical records and the literature review were combined and analyzed using SPSS version 24 (IBM Corp., Armonk, NY, USA; RRID: SCR_002865). Data normality was tested using the Shapiro–Wilk test. Categorical data were presented as frequencies and percentages, and continuous data were presented as the mean ± standard deviation (SD) or median and 25th−75th interquartile range (IQR), as appropriate according to the data distribution. Univariate analysis was performed using the 2-tailed independent *t*-test or Mann–Whitney *U*-test, as appropriate. Crosstabulation was performed using the Chi-square test or Fisher's exact test as appropriate. A *p* < 0.05 was considered statistically significant.

## Results

### Identified Patients

**Figure 1:**
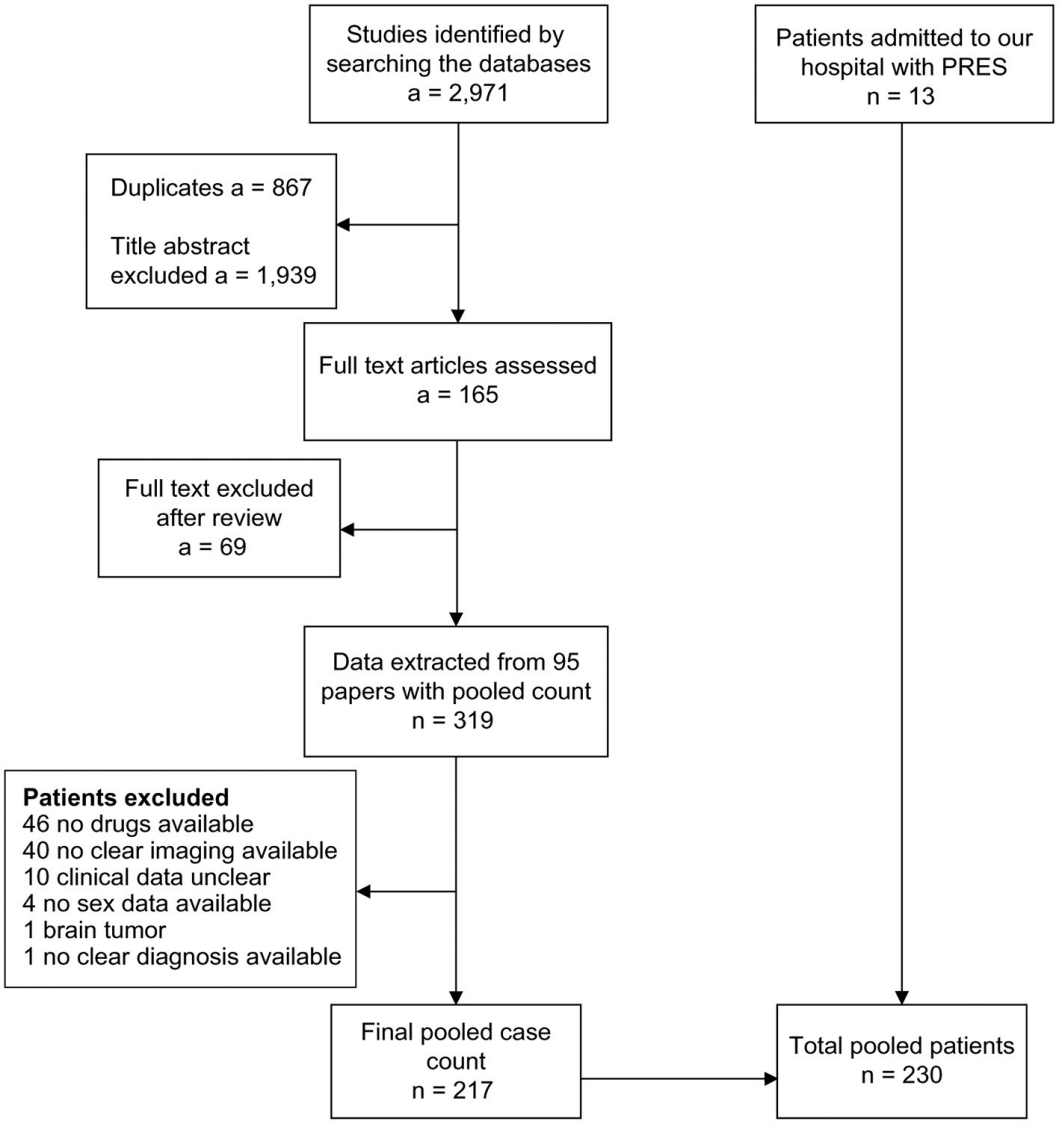
Flowchart for patient selection. a, number of articles; n, number of patients; PRES, posterior reversible encephalopathy syndrome.

Thirteen patients from our hospital and 217 patients from 74 articles indexed on PubMed, Scopus, and Web of Science met our inclusion criteria. All included articles appear in the Supplementary Material section. The flowchart of the data collected from the literature is presented in Figure 1. The clinical and MRI features of our 13 patients appear in Supplementary Table 1.

### Demographics

A total of 134 (58.3%) patients were male, with a male-to-female ratio of 1.4. The mean age at presentation was 8.7 years (range, 1.5–17.8; SD, ± 3.87; Table 1). The post-BMT group comprised 26.1% of the total patients, of which 55% were patients post-BMT for non-malignant conditions (Table 2). Patients with oncologic diseases developed PRES at a younger age than those post-BMT (mean 8.3 and 9.8 years, respectively; *p* = 0.010). The oncological disorders associated with PRES are summarized in Table 2. A total of 109 patients developed PRES during induction chemotherapy. Patients with leukemia or lymphoma were more likely to develop PRES during induction therapy than those with other oncologic diseases [99/109 (90.8%) and 10/109 (9.2%), respectively, *p* < 0.001].

**Table 1:** Comparison of demographics and clinical presentation of PRES between oncology and post-BMT patients.

**Variable**	**Oncology patients (*n* = 170)**	**Post-BMT patients (n = 60)**	**Total (n = 230)**	**P value**
Age [mean (SD)], years	8.3 (±3.77)	9.8 (±3.96)	8.7 (±3.87)	0.010
Age [median (IQR)], years	8 (5–11)	9.7 (7–12)	9 (5.65–11)	0.017
Male sex	102 (60%)	32 (53.3%)	134 (58.3%)	0.368
Male:female ratio	1.5	1.14	1.4	
**Clinical presentation**				
Seizures	142 (83.5%)	48 (80%)	190 (82.6%)	0.535
*Seizure semiology (*n* = 190)*				
FS	18 (12.7%)	2 (4.2%)	20 (10.5%)	
FIA	11 (7.7%)	1 (2.1%)	12 (6.3%)	
GS	78 (54.9%)	19 (39.6%)	97 (51.1%)	
Convulsive SE	10 (7%)	5 (10.4%)	15 (7.9%)	
NCSE	3 (2.1%)	0 (0%)	3 (1.6%)	
Not described	22 (15.5%)	21 (43.8%)	43 (22.6%)	
Headache	65 (38.2%)	25 (41.7%)	90 (39.1%)	0.640
Visual disturbances	63 (37.1%)	18 (30%)	81 (35.2%)	0.325
Altered level of consciousness	96 (56.5%)	21 (35%)	117 (50.9%)	0.004
*Number of classic clinical symptoms the patient presented with^†^*				
0	2 (1.2%)	2 (3.3%)	4 (1.7%)	
1	45 (26.5%)	20 (33.3%)	65 (28.3%)	
2	63 (37.1%)	26 (43.3%)	89 (38.7%)	
3	45 (26.5%)	8 (13.3%)	53 (23%)	
4	15 (8.8%)	4 (6.7%)	19 (8.3%)	
**Other clinical presentations** ^§^	44 (25.9%)	8 (13.3%)	52 (22.6%)	0.046
Magnesium reported	67 (39.4%)	34 (56.7%)	101 (43.9%)	0.021
Hypomagnesemia (*n* = 101)	12 (17.9%)	6 (17.6%)	18 (17.8%)	0.974
Magnesium level [mean (SD)]	0.73 (±0.20)	0.76 (±0.16)	0.74 (±0.19)	0.652
Systemic hypertension^*^ (*n* = 154)	42 (36.5%)	25 (64.1%)	67 (43.5%)	0.003
Acute hypertension (*n* = 214)	131 (82.4%)	49 (89.1%)	180 (84.1%)	0.241

*BMT, bone marrow transplantation; IQR, interquartile range; PRES, posterior reversible encephalopathy syndrome; SD, standard deviation; FS, focal seizure; FIA, focal seizure with impaired awareness; GS, generalized seizures; SE, status epilepticus; NCSE, non-convulsive status epilepticus.*

† *Classic clinical features: seizures, headache, visual disturbances, and altered level of consciousness.*

§ *Other clinical presentations: auditory changes, speech changes, hallucinations, motor changes (hemiparesis, gait disturbances), parasthesia, mood changes, body neglect.*

* *Systematic hypertension in the days preceeding PRES*.

**Table 2:** Diseases associated with PRES.

**Variable**	**Number (%)**
**Underlying oncologic diseases (*****n*** **=** **170)**	
Acute lymphoblastic leukemia	111 (65.3%)
Acute myeloid leukemia	7 (4.1%)
Other leukemias	3 (1.8%)
Non-hodgkin lymphoma	13 (7.1%)
Lymphoblastic lymphoma	3 (1.8%)
Bone tumors	4 (2.4%)
Germ cell tumors	3 (1.8%)
Neuroblastoma	11 (5.9%)
Hepatoblastoma	3 (1.8%)
Pheochromocytoma	2 (1.2%)
Hemophagocytic lymphohistiocytosis	7 (4.1%)
Wilms tumor	2 (1.2%)
Others	2 (1.2%)
*Underlying post-BMT indications (*n* = 60)*	
Malignant diseases	27 (45%)
Non-malignant diseases	33 (55%)
*Underlying post-BMT for malignant diseases (*n* = 27)*	
Relapsed ALL	6 (22.2%)
ALL with poor cytogenetics	5 (18.5%)
Relapsed AML	2 (7.4%)
AML with poor cytogenetics	4 (14.8%)
Chronic myeloid leukemia	4 (14.8%)
Ewing sarcoma (autologous BMT)	1 (3.7%)
Langerhans cell histiocytosis	2 (7.4%)
Myelodysplastic syndrome	3 (11.1%)
**Underlying post-BMT for non-malignant diseases (*****n*** **=** **33)**	
Beta thalassemia major	16 (48.5%)
Sickle cell anemia	2 (6%)
Severe aplastic anemia	8 (24.2%)
Bone marrow failure syndromes	4 (12.1%)
X-linked adrenoleukodystrophy	1 (3%)
Congenital dyserythropoietic anemia	2 (6%)

*BMT, bone marrow transplantation; PRES, posterior reversible encephalopathy syndrome; ALL, acute lymphoblastic leukemia; AML, acute myeloid leukemia*.

### Medications

The administered chemotherapy drugs appear in the Supplementary Table 2. A total of 103 (44.8%) patients received age-appropriate dosing of intrathecal (IT) chemotherapy, most commonly IT methotrexate (82/103, 79.6%), followed by triple IT chemotherapy (19/103, 18.4%) and IT cytarabine (2/103, 1.9%). Seven patients (3%) who developed PRES were not on any therapy; two were post-BMT and off IST. PRES was the first presentation of two patients with pheochromocytoma, one with neuroblastoma, and one with peripheral neuroectodermal tumor. One patient developed PRES after surgical resection of a relapsed renal cell carcinoma. The post-BMT patients received various medications depending on the timing of PRES occurence, ranging from day −18 to day +455, with the most common medications being the conditioning regimens according to disease and IST.

A total of 62 patients were on IST; the most commonly used medication was cyclosporine A (CsA). The indications for IST were post-BMT and hemophagocytic lymphohistiocytosis (HLH) in 55 (88.7%) and 7 (11.3%) cases, respectively. The CsA level was reported in only 37 patients and was toxic in 15 (40.5%).

### Clinical Presentation

The most common clinical presentation of PRES was seizures in 82.6% of patients, followed by an altered level of consciousness (50.9%) (Table 1). The most common type of seizure was generalized seizures (51.1%) followed by focal seizures (10.5%). There were no reports of patients post-BMT who developed non-convulsive status epilepticus (NCSE). Oncology patients were more likely to present with encephalopathy compared with post-BMT patients (56.5 and 35%, respectively, *p* = 0.004). Overall, 52 (22.6%) patients presented with various neurological manifestations that are not classically associated with PRES, mainly motor abnormalities (24/52, 46.2%) such as hemiparesis, weakness, gait disturbances, and dystonia. Non-typical neurologic clinical presentations occurred more in oncology patients than in post-BMT patients (25.9 and 13.3%, respectively, *p* = 0.046).

The BP status was reported in 154 patients in the days preceding the PRES episode. A total of 43.5% (66/154) of patients had a history of S-HTN before PRES, which was mainly reported for patients with acute lymphoblastic leukemia (ALL) and post-BMT. Post-BMT patients were more likely to have a history of S-HTN before the PRES episode than oncology patients (64.1 and 36.5%, respectively, *p* = 0.003). Rapid acute elevation of BP was found in 131 (88.5%) of the 148 reported patients without previously known HTN. Additionally, 101 patients had available magnesium levels, and 17.8% had hypomagnesemia. No statistical difference was recorded in the frequency of acute HTN and hypomagnesemia between post-BMT and oncology patients.

### Imaging Findings

The most commonly reported radiological abnormalities were detected in the parietal and occipital lobes (Table 3). The parietal lobe was more likely to be involved in oncology than in post-BMT patients (85.9 and 68.3%, respectively, *p* = 0.004). The radiological findings were atypical in 74.3% of cases, with no difference between oncology and post-BMT patients. The most common atypical locations were the frontal lobe (37.8%) and the temporal lobe (26.1%). Twenty-seven (11.7%) patients had isolated lesions, mainly in the occipital lobe (12/27, 44.4%). Twelve of 230 patients (5.2%) did not show parietal or occipital lobe involvement.

**Table 3:** Comparison of imaging features and neurologic outcomes of PRES between oncology and post-BMT patients.

**Variable**	**Oncology patients** **(*n* = 170)**	**Post-BMT patients** **(*n* = 60)**	**Total (*n* = 230)**	***P*-value**
**Imaging type**				
Typical	45 (26.5%)	14 (23.3%)	59 (25.7%)	0.632
Atypical	125 (73.5%)	46 (76.7%)	171 (74.3%)	
Parietal lobe	146 (85.9%)	41 (68.3%)	187 (81.3%)	0.003
Occipital lobe	142 (83.5%)	52 (86.7%)	194 (84.3%)	0.565
Frontal lobe	66 (38.8%)	21 (35%)	87 (37.8%)	0.6
Temporal lobe	45 (26.5%)	15 (25%)	60 (26.1%)	0.824
Cerebellum	27 (15.9%)	10 (16.7%)	37 (16.1%)	0.887
Brainstem	5 (2.9%)	1 (1.7%)	6 (2.6%)	NS 1
Thalamus/basal ganglia	8 (4.7%)	4 (6.7%)	12 (5.2%)	0.516
Watershed area	19 (11.2%)	4 (6.7%)	23 (10%)	0.454
Spinal cord	1 (0.6%)	1 (1.7%)	2 (0.9%)	0.455
Hemorrhage	3 (1.8%)	5 (8.3%)	8 (3.5%)	0.030
Isolated	19 (11.2%)	8 (13.3%)	27 (11.7%)	0.655
**Isolated locations (*****n*** **=** **27)**				
Parietal	7 (36.8%)	1 (12.5%)	8 (29.6%)	0.323
Occipital	7 (36.8%)	5 (62.5%)	12 (44.4%)	
Frontal	4 (21.1%)	1 (12.5%)	5 (18.5%)	
Cerebellum	1 (5.3%)	0 (0%)	1 (3.7%)	
Parasagittal	0 (0%)	1 (12.5%)	1 (3.7%)	
Total	19 (100%)	8 (100%)	27 (100%)	
Imaging repeated	126 (74.1%)	50 (50%)	156 (67.8%)	0.001
Time elapsed before follow-up MRI (weeks)	4 (IQR 2.85–8)	4 (IQR 2–14)	4 (IQR 2.1–8)	0.590
**Repeated imaging findings (*****n*** **=** **151)**				
Resolved	97 (79.5%)	19 (65.5%)	116 (76.8%)	0.091
Improved	24 (19.7%)	10 (34.5%)	34 (22.5%)	
Not resolved	1 (0.8%)	0	1 (0.7%)	
Long-term MRI changes	18 (14.8%)	6 (18.8%)	24 (15.6%)	0.579
**Outcome**				
Follow-up data available	144 (84.7%)	56 (93.3%)	200 (87%)	0.118
Duration of follow-up (months)	12 (8–29.3)	10 (IQR 2.2–35)	12 (IQR 4.75–35.2)	0.140
**Neurologic outcome (*****n*** **=** **195)**				
None	123 (86.6%)	43 (81.1%)	166 (85.1%)	0.338
Chronic epilepsy	18 (12.7%)	2 (3.8%)	20 (10.3%)	0.108
Others^†^	1 (0.7%)	8 (15.1%)	9 (4.6%)	<0.001
Recurrent	11 (6.5%)	5 (8.3%)	16 (7%)	0.570
**Mortality (*****n*** **=** **200)**				
Yes	28 (19.4%)	24 (42.9%)	52 (26%)	0.001
No	116 (80.6%)	32 (57.1%)	148 (74%)	

*BMT, bone marrow transplantation; IQR, interquartile range; MRI, magnetic resonance imaging; NS, not significant; PRES, posterior reversible encephalopathy syndrome.*

† *Other neurologic outcome: visual disturbances, sensorineural hearing loss, mental retardation, cognitive impairment, long term motor weakness, aphasia*.

Imaging was repeated in 151 patients (65.7%) after a median of 4 (IQR 2.1–8) weeks. The previously reported imaging findings resolved in 76.8% (116/151) of patients. In 11 (7.3%) cases, imaging improved on further repeated MRI, while in 24 (15.9%) chronic radiological changes were reported. The imaging findings were more likely to resolve if imaging was performed later [median 4 (IQR 3.5–8) weeks compared to 2.6 (IQR 1.4–6) weeks, *p* = 0.004]. The most common long-term MRI changes were high-intensity signals on T2-weighted imaging (6/24, 25%), followed by mesial temporal sclerosis (4/24, 16.7%) and gliosis (4/24, 16.7%).

### Electroencephalogram Findings

A total of 63 patients had reported EEG findings, of which 12 (19%) were post-BMT patients (Table 4). The most common reported abnormalities were focal slowing (20.6%) and various types of epileptiform discharges (20.6%). The EEG finding was reported as normal in 11 patients (17.5%), of which 10 (90.1%) were from the oncology group. Localization of the lesions was available only in 17 patients, of which 76.5% were described in the posterior regions and 23.5% in the temporal region. We were not able to correlate the localized EEG findings with the imaging results due to the small sample size.

**Table 4:** EEG findings of PRES between oncology and post-BMT patients.

**Variable**	**Oncology patients** **(*n* = 51)**	**Post-BMT patients** **(*n* = 12)**	**Total** **(*n* = 63)**
**EEG findings**			
FS	12 (23.5%)	1 (8.3%)	13 (20.6%)
DS	5 (9.8%)	2 (16.7%)	7 (11.1%)
LPD	4 (7.8%)	0 (0%)	4 (6.3%)
ED	11 (21.6%)	2 (16.7%)	13 (20.6%)
SE	0 (0%)	4 (33.3%)	4 (6.3%)
NCSE	2 (3.9%)	0 (0%)	2 (3.2%)
DS with ED	4 (7.8%)	2 (16.7%)	6 (9.5%)
Irregular background	1 (2%)	0 (0%)	1 (1.6%)
Post ictal	2 (3.9%)	0 (0%)	2 (3.2%)
Normal	10 (19.6%)	1 (8.3%)	11 (17.5%)
**Localization of abnormality (*****n*** **=** **17)**			
Posterior	9 (75%)	4 (80%)	13 (76.5%)
Temporal	3 (25%)	1 (20%)	4 (23.5%)
Total	12 (100%)	5 (100%)	17 (100%)

*FS, focal slowing; DS, diffuse slowing; LDP, lateralized periodic discharges; ED, epileptic discharges; SE, status epilepticus; NCSE, non-convulsive status epilepticus*.

There were 18 patients who developed clinical status epilepticus (SE) (15 convulsive and 3 non-convulsive), of which 10 had an EEG reported at unclear timings. The EEG showed SE, lateralized periodic discharges, epileptiform discharges, NCSE, and focal slowing in 40, 20, 20, 10, and 10%, respectively.

### Treatment

All patients were treated to control suspected risk factors. Complete clinical recovery was observed in 97.8% of patients (225/230). However, four patients were unable to resolve the episode and died, and the author did not elaborate on one patient's improvement prior to her death 15 days after developing PRES. Most of the patients completed chemotherapy as per the protocol, including IT chemotherapy.

Among the 62 patients on IST, neurologic outcome was described in 53 patients, of whom 7 (13.2%) had HLH. The response of the treatment centers was divided into three groups: the first group discontinued IST [15.1% (8/53) of patients], the second switched to a different agent [58.5% (31/53)], and the third group continued on the same agent with or without dose modifications [26.4% (14/53)]. The eight patients in whom IST was discontinued were post-BMT patients, and none of them experienced PRES recurrence. Of the 45 patients who continued on IST, the medication was changed in 31 (68.9%) patients. The medication was more likely to change in post-BMT than in HLH patients [29/38 (76.3%) and 2/7 (28.6%), respectively, *p* = 0.023]. Various new ISTs were initiated, including tacrolimus (10/31, 32.3%), mycophenolate mofetil (MMF) alone (8/31, 25.8%), MMF with sirolimus (9/31, 29%), and other less common combinations (4/31, 12.9%). The same IST was continued in 14 patients: one was on tacrolimus and 13 were on CsA. The patient who continued tacrolimus reported PRES recurrence. Of the 13 patients who continued treatment with CsA, recurrence was noted in 23.1%, whereas a recurrence rate of 11.1% (3/27) was found in patients who were changed to another IST; however, the difference was not statistically significant (*p* = 0.370).

Overall, 16 (7%) patients reported PRES recurrence at a median of 8 (IQR 1.6–16) weeks after the initial episode. No difference was observed in the incidence of recurrence between oncology and post-BMT patients (6.5 and 8.3%, respectively, *p* = 0.570).

### Outcome

The data regarding neurologic outcome were available for 195 patients (87%), with a median follow-up of 12 (IQR 4.7–35.2) months and a mean of 24.6 (range 0–264) months. Chronic epilepsy occurred in 10.3% (20/195) of patients in the cohort. Other rare neurologic abnormalities, which occurred in 4.6% (9/195) of patients, included cognitive impairment, long-term motor weakness, visual disturbance, and aphasia. Oncology patients were more likely to develop chronic epilepsy (12.8%, *p* = 0.108), whereas post-BMT patients were more likely to develop rare neurologic abnormalities (15.1%, *p* < 0.001). Of the patients with long-term MRI changes detected, 45.5% (10/22) developed chronic neurological abnormalities (*p* < 0.001).

The mortality rate in the cohort was 26% (52/200). Most patients died because of disease or treatment-related complications. The mortality rate was higher in post-BMT patients than in oncology patients (42.9 and 19.4%, respectively, *p* = 0.001). Among the 52 deaths, 5 (2.5%) were PRES-related and were all part of the post-BMT group. The PRES-related mortalities were secondary to non-recovery in three patients, brain herniation in one, and subarachnoid hemorrhage in one patient.

## Discussion

In this study, we described the clinical and radiological features of PRES in pediatric oncology and post-BMT patients, and investigated the risk factors associated with PRES. The oncology and post-BMT groups were similar, with the exception of age and with slight differences in clinical presentation and neuroimaging findings. Our study findings demonstrated that PRES was more common in male patients and in patients at a younger age in the oncology population. PRES was associated with S-HTN, particularly when accompanied by IST. Rare neurologic clinical presentations were more common in post-BMT patients, who were more likely to die due to PRES-related complications than in oncology patients.

Thavamani et al. reported that the mean age of PRES occurence in children was 12.54 years, with PRES being more common in adolescents (14). The median age at presentation in the oncology and post-BMT groups ranged from 6.5 to 10 years, depending on the cohort studied (5, 15–19). Our data confirmed the results reported in the literature, and showed that patients who developed PRES post-BMT were older. In adults, PRES was more common in women, even after excluding patients with eclampsia (18). Kamiya-Matsuoka et al. reported on 69 adult oncology and post-BMT patients, 52 (75%) of whom were women (20). Our study, and most pediatric oncology and post-BMT studies, have shown that PRES is more common in male patients (15–17, 21). We had previously showed that female sex was considered a risk factor in post-BMT patients; however, with a greater number of patients and an expansion of the reviewed literature, the data showed that PRES was more common in male patients (22). This may reflect the discrepancies in the literature, and more extensive studies with controlled settings are required to accurately study sex as a risk factor for PRES. A large epidemiologic study on PRES in pediatric patients showed that male sex was protective (14); however, the study did not specify patient sex per disease. Therefore, further exploration is warranted to determine whether male patients are at increased risk in the oncology and post-BMT settings.

PRES has been associated with exposure to corticosteroids, cytotoxic chemotherapy, IST, and targeted therapy (12). PRES may occur even several months after drug exposure and with serum drug concentrations within the normal range (3), such as in the cases where chemotherapy is used as a single agent or after a multi-drug regimen (12, 23). Shah-Khan et al. proposed criteria for anti-neoplastic PRES, including exposure to medications for up to 4 months before PRES (23). Several studies have implicated specific drugs as offenders; however, it is difficult to generalize these findings because most patients receive multi-drug therapies over a long period of time. It has been suggested that circulating toxins related to multiagent regimens cause direct endothelial dysfunction, potentially contributing to PRES development (12). No single chemotherapy agent or regimen has been identified as causal and consistently associated (24). Moreover, the association between PRES and the underlying biology of tumors has been considered (11). All drugs in our review have been previously reported in the adult literature. The oncology patients in our series continued their chemotherapy protocols, mostly without modifications, and only 16 (7%) developed recurrence. Most of the patients who developed recurrence were rechallenged with the same suspected causative medications, and no additional recurrences were reported.

CsA is the most studied immunosuppressive medication associated with PRES. Nephrotoxicity, neurotoxicity, and severe HTN are major side effects of CsA (25, 26). CsA neurotoxicity may be exacerbated in the presence of hypomagnesemia, hypocholesterolemia, and HTN (23, 25). In turn, CsA may further aggravate HTN through central and peripheral mechanisms (27). Regular measurements of CsA levels in the blood are essential for optimizing immunosuppressive therapy; however, neurotoxicity may occur at normal and high drug levels (25). Here, we observed variable levels of CsA associated with PRES, with a median of 223 (IQR 158–338) ng/L.

S-HTN and hypomagnesemia are common risk factors of PRES in adults and children (28–30) but were reported in <50% of the patients from the articles included in this study. The timing and severity of high BP related to the development of PRES remain unclear. Adult patients with cancer may typically experience BP increases of 10–20% from baseline (11). A case-control study with children at high risk for developing PRES found that the peak BP over the preceding 14 days was highly associated with PRES when compared to controls with similar risk factors (9). Acute HTN has been described as an independent risk factor for PRES and appears in up to 75% of pediatric cancer patients, which is consistent with our result of 84.1% (19). Regarding the variability of BP presentations, it has been proposed that acute HTN may be a result of the protective response of PRES, rather than a cause (31, 32). CsA and corticosteroids are associated with dose-dependent HTN (10). Schwartz et al. reported that the only major factor associated with the neurotoxic effects of CsA in all patients was HTN (33). In our cohort, of the 154 patients with reported BP data in the days preceding PRES, 34 (22.1%) were using IST. Patients receiving IST who developed PRES were more likely to have S-HTN, indicating the importance of BP control in patients receiving IST and the need for close monitoring of new neurological symptoms.

Numerous studies have reported a correlation between hypomagnesemia and PRES (24). The reported hypomagnesemia incidence range is 0.4–25% depending on the cohort studied (14, 19). Although magnesium levels are relevant, they were only reported in 43% of the patients included in the study. It is important to consider that any missing data in the present study may have caused bias. Magnesium levels were more likely to appear in post-BMT patients. No difference was observed in the frequency of hypomagnesemia in the oncology and post-BMT patients. This could be due to the fact that only 1% of total body magnesium is extracellular; thus, the serum magnesium levels may not reflect actual magnesium deficiency (34). Because abnormal magnesium homeostasis may be involved in seizure initiation, causing neuronal hyperexcitability (35), it may be beneficial to administer magnesium to patients with PRES, even when the magnesium levels are normal. Nevertheless, the role of magnesium prophylaxis in preventing PRES remains unclear and requires further research.

The clinical onset of PRES may be acute or subacute, with symptoms developing within a few hours or up to several days (2, 3). Seizures are reportedly the most common presentation in pediatric patients, whereas adults present more commonly present with encephalopathy (3, 13, 36). Our data were similar to those reported in the study by Zama et al. wherein seizures and encephalopathy were more common than other symptoms in pediatric oncology patients (19). PRES is a known etiology associated with SE, particularly in the presence of chemotherapy (37, 38). However, the association of NCSE with PRES has not been well-described in the literature. Bastide et al. reported NCSE in 16% of adult patients admitted to the intensive care unit with PRES that required continuous EEG monitoring (39). In our study, NCSE was reported in 1.6% of the cohort, but none of the patients were post-BMT. One patient in the cohort reported no clinical seizures, and EEG findings indicated the presence of NCSE. The duration of EEG monitoring for the patients was not defined in most cases. This is likely due to the difficulty in diagnosing NCSE, given that it is a pathology that is likely “hidden in plain sight” and requires a high index of suspicion (40). Visual disturbances have been reported in up to two thirds of adult patients, whereas these disturbances are less frequent in pediatric patients (2, 3, 36). In our study, only 35.2% of the patients reported visual changes, with no difference between the oncology and post-BMT group, potentially because children may not complain of visual field defects or parents may disregard mild complaints. This emphasizes the importance of educating parents and older children to elaborate on any unusual symptoms. Moreover, patients may present with neurological abnormalities other than those reported typically, such as paresis, psychosis, and auditory abnormalities (19, 32). This pattern was more common in oncology patients than in post-BMT patients, particularly in those with ALL during induction chemotherapy. The differences in clinical presentation may suggest different mechanisms of PRES development owing to chemotherapy and IST.

There is no conclusive evidence supporting a correlation between the different clinical presentations and the affected brain regions assessed through MRI (41). In our cohort, 192 patients had occipital lobe lesions; however, only 69 (35.9%) had visual disturbances. We found atypical imaging findings on MRI in 74.3%, similar to previous reports on pediatric patients, with no significant between-group differences (8, 16). The frequency range of atypical presentations is 10–58% in adult patients (8). Here, 5.2% (12/230) of the patients did not have parietal or occipital lobe involvement. MRI changes were isolated in 11.7% (27/230) of patients, particularly to the occipital lobe. Identifying atypical PRES imaging findings is vital to avoid delays in the diagnosis and treatment that may affect neurologic outcomes (42).

Intracranial hemorrhage is a complication observed on imaging in 8–28% of pediatric PRES cases (12). It is reportedly more common in post-BMT than in post-solid organ transplantation patients, potentially because of the underlying coagulopathy post-BMT patients (42). Hemorrhages were reported in 3.5% (8/230) of patients, five of whom were post-BMT. Hemorrhage was more common in the post-BMT than in the oncology group (*p* = 0.030). Saad et al. found no difference in the incidence of hemorrhage among patients with normal, moderately elevated, or severely elevated BP (42). However, in our cohort, all patients with hemorrhage had S-HTN (*p* = 0.001). This may indicate the increased susceptibility of pediatric patients to the effects of elevated BP in the presence of endothelial injury.

EEG is a commonly used assessment tool in patients with encephalopathy and seizures. Specific EEG patterns have been associated with various conditions, such triphasic waves in toxic/metabolic abnormalities and the extreme delta brush pattern in autoimmune encephalitis (43). However, to the best of our knowledge, no specific pattern has been consistently reported for EEGs in patients with PRES, and the reported origin of seizures on EEG may not correlate with MRI findings (43, 44). In a study conducted on adult oncology patients with PRES, the most common EEG finding on a standard recording was diffuse slowing (44). Grioni et al. described video EEG recordings of ten patients with PRES with diffuse slowing and focal unilateral temporo-occipital activity (45). A cohort of critically ill adults with PRES on continuous EEG monitoring reported that 62% had non-convulsive seizures or periodic discharges with 74% localized to the posterior region (39). In our study, we found the most common findings were focal slowing and various forms of epileptiform discharges, and the abnormalities were most commonly localized to the posterior region of the brain. The different EEG findings can be related to the duration of EEG, given that standard recording compared to continuous EEG recording may not be sufficient to detect findings. A study of continuous EEG monitoring on a large sample size would help determine if any specific pattern can be identified.

No specific treatment exists for PRES; it is primarily managed with supportive care (2, 3, 12), including BP control, correction of metabolic abnormalities such as hypomagnesemia, and anticonvulsant medications (12). In oncology, the offending drug is not usually directly determined, and drug modification is associated with an increased risk of relapse. Most patients in our cohort continued their chemotherapy protocol, with a recurrence rate of 7.1%.

There is controversy regarding whether changing IST drug or continuing with the same medication with or without dose modification is the optimal management to reduce the risk of recurrence. It has been reported that the recurrence rates do not differ regardless of IST management (46–48). Hammerstrom et al. reported no difference in the discharge outcome related to the management strategy used for tacrolimus (continuing or changing to another agent); however, they did not elaborate on the recurrence risk (46). Our data showed no difference was recorded in recurrence between patients who continued the same IST and those who changed to another IST medication.

The typical hallmark of PRES is the reversibility of the clinical picture and imaging findings. The presence of irreversible cases poses challenges. Clinical recovery usually occurs earlier than neuroimaging resolution (31). Our data confirm this finding; the later the MRI was performed, the more likely it was to observe complete resolution. Furthermore, PRES has been associated with recurrent seizures in 2.4–8.3% of cases (48, 49). Future seizures episodes may indicate PRES recurrence or chronic epilepsy development. In our study, PRES recurrence was reported in 7% of patients, whereas epilepsy occurred in 10.3%. This may not represent the actual incidence of epilepsy development. The data on chronic epilepsy may have been inflated because they were collected from case reports and case series; which are studies that may focus more on unusual presentations or epilepsy cases. Epilepsy may develop after the patient follow-up ends, and it has been reported to occur up to 7 years after PRES (3, 50). Therefore, we could not draw any conclusions on the incidence of epilepsy in our cohort. The patients with oncologic diseases and post-BMT have multiple risk factors for developing epilepsy, including previous chemotherapy with multiple modes of administration and radiation. This causes further difficulty in drawing conclusions to implicate PRES as a sole risk factor. Therefore, further studies are warranted on the development of chronic neurologic complications in oncology and post-BMT patients in a control group compared to patients who developed PRES.

PRES recurrence has been reported to occur in 3.8–12.5% of patients, which is in line with our findings (13, 51). PRES-related mortality has been reported in 2.6–3.2% of children and 3–6% of adults (3, 14, 52). PRES-related mortality was 2.5%, all of which in the post-BMT group. This was consistent with reports indicating that post-transplantation was a predictor of poor outcome in adult patients with PRES (53).

This study has some limitations. First, this was a retrospective single-center study reviewing a series of case reports available in the literature, and it did not include a control group; this may have introduced a selection bias. Moreover, the study focused on examining the association between risk factors and PRES rather than identifying risk factors contributing to PRES development. The case reporting may have been biased because some articles may have reported cases of PRES in patients with specific diseases, presentations, or outcomes of certain complications. There were excluded papers that mentioned the suspected offending drug without details of other drugs the patient was exposed to, in an effort to decrease the bias in the number of causative drugs. However, it may have also caused underestimation of the importance of these drugs in absence of large studies. Several studies on large cohorts were excluded according to our study criteria because they reported summary data and not details of individual patients. Further, the different medical specialties reporting the disease may lead to different angles of exploration of the cases. In our study, neuroimaging timings and treatment protocols were not standardized across and within the included studies. There was a lack of data consistency for long-term clinical imaging findings, recurrence, and cognitive outcomes. Nevertheless, we selected patients described in the literature whose detailed data matched the data collected from our center, aiming to amass a large number of cases reflecting the clinical and radiological PRES features and to identify potential risk factors. A case-control study evaluating the details of drugs and clinical features, with standardized timing of initial and follow-up imaging, and uniform management is needed to elicit the risk factors appropriately.

PRES is increasingly recognized in pediatric oncology and post-BMT patients. Physicians should be aware of the variability in clinical presentation and retain a high index of suspicion in the presence of risk factors such as chemotherapy, IST, S-HTN, and hypomagnesemia. Most imaging findings in pediatric patients were atypical, occurring even without the involvement of the parietal or occipital lobe, which should not hinder the diagnosis. Supra-therapeutic levels and previous exposure to IST may not increase the risk, and changing the IST is not protective against recurrence. PRES is generally reversible, but long-term complications may occur. Here, we found that chronic epilepsy was more common in oncology patients, while rare neurologic complications were more common post-BMT.

In conclusion, our data indicated that PRES is more common in male patients, and in younger patients in oncology populations. PRES was associated with S-HTN, particularly when accompanied by IST. The difference in epilepsy incidence between the groups may indicate a different causative pathophysiology; further research is warranted, particularly regarding the duration of antiepileptic therapy. Moreover, the risk factors and standardized management may be better elucidated by a more extensive prospective study.

## Data Availability Statement

The raw data supporting the conclusions of this article will be made available by the authors, without undue reservation.

## Ethics Statement

The studies involving human participants were reviewed and approved by Institutional Review Board of King Fahad Specialist Hospital in Dammam (IRB-ONC0312). The Ethics Committee waived the requirement of written informed consent for participation. Written informed consent was not obtained from the individual(s) for the publication of any potentially identifiable images or data included in this article.

## Author Contributions

HS contributed to the conception, study design, literature review, data analysis, writing of the initial draft, and finalization of the manuscript. SAla contributed to the conception, study design, and review of the final draft. HO contributed to the study design, data revision, and writing of the initial draft. SAld critically reviewed the manuscript and contributed to the finalization of the manuscript. All authors contributed to the article and approved the submitted version.

## Conflict of Interest

The authors declare that the research was conducted in the absence of any commercial or financial relationships that could be construed as a potential conflict of interest.

## Publisher's Note

All claims expressed in this article are solely those of the authors and do not necessarily represent those of their affiliated organizations, or those of the publisher, the editors and the reviewers. Any product that may be evaluated in this article, or claim that may be made by its manufacturer, is not guaranteed or endorsed by the publisher.

## Abbreviations

ALL: acute lymphoblastic leukemia
BMT: bone marrow transplantation
BP: blood pressure
CNS: central nervous system
CPM: cyclophosphamide
CsA: cyclosporine A
EEG: electroencephalogram
F: female
HLH: hemophagocytic lymphohistiocytosis
HTN: hypertension
HypoMg: hypomagnesemia
IQR: interquartile range
IST: immunosuppressants
IT: intrathecal
IV: intravenous
LOC: level of consciousness
M: male
MMF: mycophenolate mofetil
MRI: magnetic resonance imaging
MTX: methotrexate
NCSE: non-convulsive status epilepticus
NS: not significant
Ph: Philadelphia chromosome
PRES: posterior reversible encephalopathy syndrome
S-HTN: systemic hypertension
SD: standard deviation
SE: status epilepticus.
