# Supplementary material for: Different Clinicoradiological Characteristics of Posterior Reversible Encephalopathy Syndrome in Pediatric Oncology and Post-Bone Marrow Transplantation Cases: A Retrospective Study
Source: Front Neurol. 2022 May 6;13:836033. doi: 10.3389/fneur.2022.836033 (PMC9120546; doi:10.3389/fneur.2022.836033)
Supplement: Supplementary file 1 [file Data_Sheet_1.PDF]

Supplementary Material

1 Supplementary Data

References for case reports and series that met the inclusion criteria: [1-74]

1. Aboian MS, Junna MR, Krecke KN, Wirrell EC. Mesial temporal sclerosis after posterior reversible encephalopathy syndrome. Pediatr Neurol (2009) 41:226-8. doi:10.1016/j.pediatrneurol.2009.03.007.

2. Adamczewska A, Adamczewska K, Jończyk-Potoczna K, Pieczonka A, Derwich K. Posterior reversible encephalopathy syndrome (PRES) induced by intrathecal methotrexate administration in a patient with acute lymphoblastic leukaemia. Oncol Clin Pract (2016) 12:102-4.

3. Appachu MS, Purohit S, Lakshmaiah KC, Kumari BS, Appaji L. Posterior reversible encephalopathy syndrome in pediatric acute leukemia: case series and literature review. Indian J Med Paediatr Oncol (2014) 35:79-82. doi:10.4103/0971-5851.133727.

4. Aureli V, Giammattei L, Maduri R, Daniel RT, Messerer M. Posterior reversible encephalopathy syndrome (PRES) due to neuroblastoma in a child presenting with acute hydrocephalus. Childs Nerv Syst (2018) 34:15-7. doi:10.1007/s00381-017-3640-6.

5. Balaji R, Ramachandran K, Nair M, Kusumakumari P. Chemotherapy-induced posterior reversible encephalopathy syndrome in childhood chronic myeloid leukemia. A case report and review of the literature. Neuroradiol J (2009) 22:204-8.

6. Banerjee JS, Heyman M, Palomäki M, et al. Posterior reversible encephalopathy syndrome: risk factors and impact on the outcome in children with acute lymphoblastic leukemia treated with Nordic protocols. J Pediatr Hematol Oncol (2018) 40:e13-8.

7. Baytan B, Özdemir O, Demirkaya M, Evim MS, Gunes AM. Reversible posterior leukoencephalopathy induced by cancer chemotherapy. Pediatr Neurol (2010) 43:197-201. doi:10.1016/j.pediatrneurol.2010.05.005.

8. Belaramani KM, Lai V, Li CH, Lee AC, Kwong NS. Reversible posterior leukoencephalopathy syndrome in Chinese children induced by chemotherapy: a review of five cases. Hong Kong Med J (2011) 17:61-6.

9. Bhunia N, Abu-Arja R, Bajwa RPS, Auletta JJ, Rangarajan HG. Successful treatment with eculizumab for posterior reversible encephalopathy syndrome due to underlying transplant-associated thrombotic microangiopathy in patients transplanted for sickle cell disease. Pediatr Blood Cancer (2019) 66:e27912. doi:10.1002/pbc.27912.

10. Chamdine OM, Shatila A-RS, Yassin NA, Muwakkit SA, Ibrahim AO. Cyclosporine-induced PRES in a child recipient of allogeneic bone marrow transplantation. J Pediatr Neurol (2012) 10:313-6.

11. Chan AK, Bhargava R, Desai S, Joffe A. Reversible posterior leukoencephalopathy syndrome in a child with cerebral X-linked adrenoleukodystrophy treated with cyclosporine after bone marrow transplantation. J Inherit Metab Dis (2003) 26:527-36.

12. Chaudhary N, Majeed R, Borker A. Isolated cerebellar involvement in posterior reversible encephalopathy syndrome in a child with acute lymphoblastic leukemia. Indian J Med Paediatr Oncol (2011) 32:211-3.

13. Chen TH, Chiou SS, Lin WC, et al. Posterior reversible encephalopathy syndrome in critically ill children: a case series. Intensive Care Med (2013) 39:155-6. doi:10.1007/s00134-012-2705-y.

14. Cooney MJ, Bradley WG, Symko SC, Patel ST, Groncy PK. Hypertensive encephalopathy: complication in children treated for myeloproliferative disorders—report of three cases. Radiology (2000) 214:711-6.

15. D'Angelo P, Farruggia P, Lo Bello AL, et al. Reversible posterior leukoencephalopathy syndrome: report of 2 simultaneous cases in children. J Pediatr Hematol Oncol (2006) 28:177-81.

16. Danhofer P, Tomečková M, Černá D, et al. Prognostic factors and seizure outcome in posterior reversible encephalopathy syndrome (PRES) in children with hematological malignancies and bone marrow failure: A retrospective monocentric study. Seizure (2019) 72:1-10. doi:10.1016/j.seizure.2019.08.007.

17. de Laat P, te Winkel ML, Devos AS, Catsman-Berrevoets CE, Pieters R, van den Heuvel-Eibrink MM. Posterior reversible encephalopathy syndrome in childhood cancer. Ann Oncol (2011) 22:472-8. doi:10.1093/annonc/mdq382.

18. Donmez FY, Agildere AM. Recurrent childhood PRES. Neurol Sci (2015) 36:1603-9.

19. Endo A, Fuchigami T, Hasegawa M, et al. Posterior reversible encephalopathy syndrome in childhood: report of four cases and review of the literature. Pediatr Emerg Care (2012) 28:153-7.

20. Eroglu N, Bahadir A, Erduran E. A case of ALL developing posterior reversible encephalopathy secondary to hyponatremia. J Pediatr Hematol Oncol (2017) 39:e476-8.

21. Fong CY, Hlaing CS, Aye AMM, Tay CG, Ariffin H, Ong LC. Posterior reversible encephalopathy syndrome: Malaysian haemato-oncological paediatric case series. Neurol Asia (2015) 20.

22. Fraint E, Miller R, Walter A. Posterior reversible encephalopathy syndrome and cerebral sinus thrombosis in a case of pediatric B-cell ALL. J Pediatr Hematol Oncol (2017) 39:e71-3.

23. Freedman D, Koram A, Gillson N, Aylward SC. Pediatric posterior reversible encephalopathy syndrome (PRES) with spinal cord involvement due to pheochromocytoma. Pediatr Neurol (2017) 77:92-3. doi:10.1016/j.pediatrneurol.2017.06.016.

24. Fukuyama T, Tanaka M, Nakazawa Y, et al. Prophylactic treatment for hypertension and seizure in a case of allogeneic hematopoietic stem cell transplantation after posterior reversible encephalopathy syndrome. Pediatr Transplant (2011) 15:E169-73. doi:10.1111/j.1399-3046.2010.01358.x.

25. Cho SG, Moon H, Lee JH, Lee SY, Kim CC, Lee KS. Behenoyl cytarabine-associated reversible encephalopathy in a patient with acute myelogenous leukemia. J Korean Med Sci (1999) 14:89-92.

26. Gupta A, Swaroop C, Rastogi R, Garg R, Bakhshi S. Simultaneous occurrence of posterior reversible leukoencephalopathy syndrome in two cases of childhood acute lymphoblastic leukemia induction chemotherapy. Pediatr Hematol Oncol (2008) 25:351-8. doi:10.1080/08880010802016052.

27. He MS, Yen PS, Chu SY, Lee YC. Relapsing reversible posterior leukoencephalopathy syndrome. Eye (Lond) (2006) 20:1397-9. doi:10.1038/sj.eye.6702246.

28. Heo S, Cho HJ, Jeon IS. A case of posterior reversible encephalopathy syndrome in a child with myelodysplastic syndrome following allogenic bone marrow transplantation. Pediatr Hematol Oncol (2010) 27:59-64. doi:10.3109/08880010903420661.

29. Higman MA, Port JD, Beauchamp NJ, Chen AR. Reversible leukoencephalopathy associated with re-infusion of DMSO preserved stem cells. Bone Marrow Transplant (2000) 26:797-800.

30. Honkaniemi J, Kähärä V, Dastidar P, et al. Reversible posterior leukoencephalopathy after combination chemotherapy. Neuroradiology (2000) 42:895-9.

31. Hourani R, Abboud M, Hourani M, Khalifeh H, Muwakkit S. L-asparaginase-induced posterior reversible encephalopathy syndrome during acute lymphoblastic leukemia treatment in children. Neuropediatrics (2008) 39:46-50. doi:10.1055/s-2008-1076740.

32. Isoda T, Mitsuiki N, Ohkawa T, et al. Irreversible leukoencephalopathy after reduced-intensity stem cell transplantation in a dyskeratosis congenita patient with TINF2 mutation. J Pediatr Hematol Oncol (2013) 35:e178-82.

33. Wan Jamaludin W, Mohd Zaki F, Tumian N, Wong C, Abdul Wahid S. Posterior reversible encephalopathy syndrome as the initial presentation of acute lymphoblastic leukaemia. J Hematol Malig (2011) 1.

34. Jennane S, Mahtat el M, Konopacki J, et al. Cyclosporine-related posterior reversible encephalopathy syndrome after cord blood stem cell transplantation. Hematol Oncol Stem Cell Ther (2013) 6:71. doi:10.1016/j.hemonc.2013.05.002.

35. Kaito E, Terae S, Kobayashi R, Kudo K, Tha KK, Miyasaka K. The role of tumor lysis in reversible posterior leukoencephalopathy syndrome. Pediatr Radiol (2005) 35:722-7. doi:10.1007/s00247-005-1434-6.

36. Kapoor R, Simalti A, Kumar R, et al. PRES in pediatric HSCT: a single-center experience. J Pediatr Hematol Oncol (2018) 40:433-7.

37. Kartal Ö, Gürsel O, Ünay B, Arda KN, Atas E. Posterior reversible encephalopathy syndrome in a normotensive child after allogeneic hematopoietic stem cell transplantation. P R Health Sci J (2019) 38:272-4.

38. Kawamura Y, Ohashi M, Asahito H, Takahashi Y, Kojima S, Yoshikawa T. Posterior reversible encephalopathy syndrome in a child with post-transplant HHV-6B encephalitis. Bone Marrow Transplant (2012) 47:1381-2. doi:10.1038/bmt.2012.42.

39. Kheir JN, Lawlor MW, Ahn ES, et al. Neuropathology of a fatal case of posterior reversible encephalopathy syndrome. Pediatr Dev Pathol (2010) 13:397-403. doi:10.2350/09-04-0634-CR.1.

40. Kushner BH, Modak S, Basu EM, Roberts SS, Kramer K, Cheung NK. Posterior reversible encephalopathy syndrome in neuroblastoma patients receiving anti-GD23F8 monoclonal antibody. Cancer (2013) 119:2789-95. doi:10.1002/cncr.28137.

41. Lai CC, Chen SJ, Lien SH, Lo CP, Cheng SN. Posterior reversible encephalopathy in a child with Langerhans cell histiocytosis following allogeneic PBSCT treatment with cyclosporine. Eur J Pediatr (2008) 167:817-20.

42. Lee G, Lee SE, Ryu KH, Yoo ES. Posterior reversible encephalopathy syndrome in pediatric patients undergoing treatment for hemophagocytic lymphohistiocytosis: clinical outcomes and putative risk factors. Blood Res (2013) 48:258-65. doi:10.5045/br.2013.48.4.258.

43. Levy CF, Oo KZ, Fireman F, et al. Reversible posterior leukoencephalopathy syndrome in a child treated with bevacizumab. Pediatr Blood Cancer (2009) 52:669-71. doi:10.1002/pbc.21866.

44. Lim YJ, Kim HJ, Lee YJ, Seol IJ, Lee YH. Clinical features of encephalopathy in children with cancer requiring cranial magnetic resonance imaging. Pediatr Neurol (2011) 44:433-8. doi:10.1016/j.pediatrneurol.2011.01.007.

45. Lucchini G, Grioni D, Colombini A, et al. Encephalopathy syndrome in children with hemato-oncological disorders is not always posterior and reversible. Pediatr Blood Cancer (2008) 51:629-33.

46. Maher OM, Marco SA, Sadanandan S, Fireman F, Sedrak A. Retroperitoneal ganglioneuroma and reversible posterior leukoencephalopathy in a child with acute lymphoblastic leukemia. J Pediatr Hematol Oncol (2014) 36:665-6.

47. Malbora B, Avcı Z, Dönmez F, et al. Posterior reversible leukoencephalopathy syndrome in children with hematologic disorders. Turk J Haematol (2010) 27:168-76.

48. Manchana T, Sirisabya N, Lertkhachonsuk R, Tresukosol D. Transient cortical blindness during chemotherapy (PVB) for ovarian germ cell tumor. J Med Assoc Thai (2006) 89:1265-8.

49. Moorthy S, Subramaniam T, Prabhu N, Sree KK, Nair R. Posterior reversible encephalopathy syndrome in a child with pheochromocytoma. Indian J Rad Imaging (2002) 12:321.

50. Mori A, Tanaka J, Kobayashi S, et al. Fatal cerebral hemorrhage associated with cyclosporin-A/FK506-related encephalopathy after allogeneic bone marrow transplantation. Ann Hematol (2000) 79:588-92. doi:10.1007/s002770000192.

51. Morris EB, Laningham FH, Sandlund JT, Khan RB. Posterior reversible encephalopathy syndrome in children with cancer. Pediatr Blood Cancer (2007) 48:152-9. doi:10.1002/pbc.20703.

52. Muro VL, Yip S, Huh L, Connolly MB. Status epilepticus amauroticus and posterior reversible encephalopathy syndrome in children. J Clin Neurophysiol (2013) 30:344-7. doi:10.1097/WNP.0b013e31829de004.

53. Musioł K, Waz S, Boroń M, et al. PRES in the course of hemato-oncological treatment in children. Childs Nerv Syst (2018) 34:691-9. doi:10.1007/s00381-017-3664-y.

54. Navarro CE, Rodríguez PJ, Espitia OM. Fludarabine-induced posterior reversible encephalopathy syndrome in a pediatric patient with β-thalassemia: case report and literature review. Clin Neuropharmacol (2018) 41:224-9. doi:10.1097/WNF.0000000000000309.

55. Nguyen L, Crawford JR. Posterior reversible encephalopathy syndrome mimicking tumor recurrence in a patient with multiply recurrent neuroblastoma. Pediatr Neurol (2020) 110:92-4. doi:10.1016/j.pediatrneurol.2020.04.015.

56. Niyadurupola N, Burnett CA, Allen LE. Reversible posterior leucoencephalopathy syndrome: a cause of temporary cortical blindness. Br J Ophthalmol (2005) 89:924-5.

57. Noè A, Cappelli B, Biffi A, et al. High incidence of severe cyclosporine neurotoxicity in children affected by haemoglobinopaties undergoing myeloablative haematopoietic stem cell transplantation: early diagnosis and prompt intervention ameliorates neurological outcome. Ital J Pediatr (2010) 36:14.

58. Norman JK, Parke JT, Wilson DA, McNall-Knapp RY. Reversible posterior leukoencephalopathy syndrome in children undergoing induction therapy for acute lymphoblastic leukemia. Pediatr Blood Cancer (2007) 49:198-203.

59. Ozyurek H, Oguz G, Ozen S, et al. Reversible posterior leukoencephalopathy syndrome: report of three cases. J Child Neurol (2005) 20:990-3.

60. Panis B, Vlaar AM, van Well GTJ, et al. Posterior reversible encephalopathy syndrome in paediatric leukaemia. Eur J Paediatr Neurol (2010) 14:539-45.

61. Piątkowska M, Wysocki M, Styczyński J. Cerebral complications induced by calcineurin inhibitors in children after haematopoietic stem cell transplantation. wo (2011) 3:159-63.

62. Rathi B, Azad RK, Vasudha N, Hissaria P, Sawlani V, Gupta RK. L-asparaginase-induced reversible posterior leukoencephalopathy syndrome in a child with acute lymphoblastic leukemia. Pediatr Neurosurg (2002) 37:203-5.

63. Sánchez-Carpintero R, Narbona J, López de Mesa R, Arbizu J, Sierrasesúmaga L. Transient posterior encephalopathy induced by chemotherapy in children. Pediatr Neurol (2001) 24:145-8.

64. Shimizu Y, Tha KK, Iguchi A, et al. Isolated posterior fossa involvement in posterior reversible encephalopathy syndrome. Neuroradiol J (2013) 26:514-9.

65. Shin RK, Stern JW, Janss AJ, Hunter JV, Liu GT. Reversible posterior leukoencephalopathy during the treatment of acute lymphoblastic leukemia. Neurology (2001) 56:388-91.

66. Shkalim-Zemer V, Konen O, Levinsky Y, et al. Calcineurin inhibitor-free strategies for prophylaxis and treatment of GVHD in children with posterior reversible encephalopathy syndrome after stem cell transplantation. Pediatr Blood Cancer (2017) 64:e26531.

67. Stott VL, Hurrell MA, Anderson TJ. Reversible posterior leukoencephalopathy syndrome: a misnomer reviewed. Intern Med J (2005) 35:83-90.

68. Suminoe A, Matsuzaki A, Kira R, et al. Reversible posterior leukoencephalopathy syndrome in children with cancers. J Pediatr Hematol Oncol (2003) 25:236-9.

69. Suzuki D, Kobayashi R, Iguchi A, et al. Tumor lysis syndrome as a risk factor for posterior reversible encephalopathy syndrome in children with hematological malignancies. Int J Hematol (2014) 100:485-9. doi:10.1007/s12185-014-1658-z.

70. Tambasco N, Mastrodicasa E, Salvatori C, et al. Prognostic factors in children with PRES and hematologic diseases. Acta Neurol Scand (2016) 134:474-83. doi:10.1111/ane.12570.

71. Tang JH, Tian JM, Sheng M, et al. Study of posterior reversible encephalopathy syndrome in children with acute lymphoblastic leukemia after induction chemotherapy. J Child Neurol (2016) 31:279-84.

72. Tavil B, Isgandarova F, Bayhan T, et al. Sorafenib-induced posterior reversible encephalopathy syndrome in a child with FLT3-ITD-positive acute myeloid leukemia. J Pediatr Hematol Oncol (2016) 38:240-2.

73. Teive HA, Brandi IV, Camargo CH, et al. Reversible posterior leucoencephalopathy syndrome associated with bone marrow transplantation. Arq Neuropsiquiatr (2001) 59:784-9.

74. Zhang R, Jin L, Cheng H, et al. Reversible posterior leukoencephalopathy syndrome sometimes could be irreversible: a case following tumor lysis syndrome in childhood Burkitt’s lymphoma. Chin Med J (Engl) (2016) 129:480-3.

2 Supplementary Tables

Supplementary Table 1. Demographic, clinical, and radiologic features, and neurologic outcomes of patients with PRES in our hospital.

Case	Age (year) / sex	Diagnosis	Medications	Symptoms	Systemic HTN<br><br>Acute HTN	HypoMg	Location (lobes)	Imaging repeated<br><br>Duration<br><br>Results	Recurrence	Follow-up duration<br><br>Outcome
**1**	12 / M	Post-BMT (Fanconi anemia, day -4)	Conditioning (fludarabine, CPM)<br>IST (CsA)	Seizures	No<br><br>Yes	Yes	Parietal<br>Occipital<br>Temporal<br>Cerebellum	Yes<br><br>4 weeks<br><br>Resolved	No	4 months<br><br>Death (non PRES-related)
**2**	12 / F	Post-BMT (Ph-positive ALL, day +144)	IST (CsA and MMF)<br>Imatinib<br>Rituximab<br>Steroids	Seizures<br>Visual changes<br>Altered LOC	No<br><br>Yes	Yes	Parietal<br>Occipital	No	No	84 months<br><br>Normal
**3**	10 / M	T-cell ALL, CNS negative	Cytarabine<br>Vincristine<br>PEG-asparaginase<br>CPM<br>6-mercaptopurine	Seizures	No<br><br>Yes	Yes	Parietal<br>Occipital<br>Frontal	Yes<br><br>2.6 weeks<br><br>Improved Second MRI at 8 weeks showed	No	96 months<br><br>Normal

								residual<br>high signal<br>intensity		
**4**	16 / F	B-cell ALL,<br>CNS<br>negative	IV high dose<br>MTX<br>IT MTX<br>Vincristine<br>6-<br>mercaptopurine	Seizures<br>Headache<br>Altered LOC	Yes<br><br>Yes	Yes	Parietal<br>Temporal<br>Cerebellum<br>Thalamus<br>Basal<br>ganglia	Yes<br><br>7 weeks<br><br>Resolved	No	82<br>months<br><br>Chronic<br>epilepsy
**5**	8 / F	B-cell ALL<br>(Ph-<br>positive),<br>CNS<br>negative	Triple IT (MTX,<br>cytarabine,<br>hydrocortisone)<br>PEG-<br>asparaginase<br>Doxorubicin<br>CPM<br>Imatinib	Seizures	No<br><br>No	No	Parietal<br>Occipital	No	No	3 months<br><br>Death<br>(non<br>PRES-<br>related)
**6**	9 / F	Post-BMT<br>(severe<br>aplastic<br>anemia, day<br>+90)	IST (tacrolimus)	Seizures<br>Headache	No<br><br>No	Yes	Parietal	Yes<br><br>2 weeks<br><br>Resolved	No	67<br>months<br><br>Normal
**7**	9 / M	Burkitt<br>lymphoma	Steroids<br>IV MTX<br>IT MTX<br>Vincristine<br>CPM<br>Rituximab	Seizures<br>Headache<br>Visual<br>changes<br>Visual<br>hallucinations<br>Altered LOC	No<br><br>Yes	No	Parietal	Yes<br><br>3 weeks<br><br>Resolved	No	43<br>months<br><br>Normal
**8**	7 / F	Post-BMT<br>(beta	IST (CsA)	Seizures<br>Visual<br>changes	No<br><br>Yes	No	Parietal<br>Occipital<br>Temporal	No	No	3 months

		thalassemia, day +27)		Visual hallucinations<br>Weakness						Death (non PRES-related)
**9**	10 / F	Fanconi anemia with acute myeloid leukemia	Cytarabine<br>Clofarabine	Seizures<br>Headache	No<br><br>Yes	No	Parietal<br>Occipital<br>Temporal<br>Thalamus / Basal ganglia	Yes<br><br>3 weeks<br><br>Resolved	Yes<br><br>Occurred 14 weeks after the first episode during BMT day +4	AML relapse at 5 months<br><br>Death (Non PRES-related)
**10**	10 / M	T-cell ALL, CNS negative	Steroids<br>IT MTX<br>PEG-asparaginase<br>Vincristine<br>Doxorubicin	Seizures<br>Visual changes<br>Altered LOC	No<br><br>No	No	Parietal<br>Occipital	Not repeated between attacks<br><br>Repeated after second attack, resolved	Yes<br><br>2.6 weeks after the first episode	68 months<br><br>Chronic epilepsy
**11**	5 / F	Post-BMT (beta thalassemia, day +9)	Conditioning (CPM)<br>IST (CsA)	Seizures<br>Altered LOC	Yes<br><br>Yes	No	Parietal<br>Occipital<br>Temporal	Not repeated between attacks<br><br>Repeated after the second	Yes<br><br>3.1 weeks after the first episode	48 months<br><br>Normal

								attack, resolved		
**12**	16 / M	Post-BMT (Severe aplastic anemia, day +57)	IST (CsA) Steroids	Seizures	Yes<br><br>Yes	No	Cerebellum Parietal Occipital Temporal Frontal Petechial hemorrhage	Yes<br><br>10 days<br><br>Improved	No	1 month<br><br>Death (non PRES-related)
**13**	7 / M	Burkitt lymphoma	CPM Vincristine Steroids	Altered LOC Seizure Visual disturbances	Yes<br><br>Yes	Yes	Parietal Occipital Frontal Cerebellum Temporal	No	No	0.75 months<br><br>Death (non PRES - related)

ALL=acute lymphoblastic leukemia; BMT=bone marrow transplantation; CNS=central nervous system; CPM=cyclophosphamide; CsA=cyclosporine A; F=female; HTN=hypertension; HypoMg= hypomagnesemia; IST=immunosuppression therapy; IT=intrathecal; IV=intravenous; LOC=level of consciousness; M=male; MMF=mycophenolate mofetil;MRI=magentic resonance imaging; MTX=methotrexate; Ph=Philadelphia chromosome; PRES=posterior reversible encephalopathy syndrome

Supplementary Table 2. Medications associated with PRES

**Steroids**	**142 (61.7%)**
**Intrathecal chemotherapy**	**103 (44.8%)**
**IV methotrexate**	**22 (9.6%)**
**Ara-C**	**21 (9.1%)**
**Asparaginase**	**75 (32.6%)**
**Vincristine**	**119 (51.7%)**
**Anthracyclines**	**83 (36.1%)**
**Alkylating agents**	**32 (13.9%)**
**Platins**	**9 (3.9%)**
**Conditioning**	**10 (4.3%)**
**Immunosuppression**	**62 (27%)**
**Etoposide**	**15 (6.5%)**
**Bleomycin**	**4 (1.7%)**
**Rituximab**	**4 (1.7%)**
**Anti-GD2**	**6 (2.6%)**
**Radiation**	**2 (0.9%)**
**No treatment**	**7 (3%)**
**Others**	**22 (9.6%)**

PRES= posterior reversible encephalopathy syndrome; IV=intravenous; ARA-C=cytosine arabinoside; Anti-GD2=anti-disialoganglioside monoclonal antibodies
